# Analysis of the Hardness of Soft Relining Materials for Removable Dentures

**DOI:** 10.3390/ijerph18189491

**Published:** 2021-09-08

**Authors:** Ewa Białożyt-Bujak, Magdalena Wyszyńska, Grzegorz Chladek, Aleksandra Czelakowska, Andrzej Gala, Magdalena Orczykowska, Agata Białożyt, Jacek Kasperski, Małgorzata Skucha-Nowak

**Affiliations:** 1 Department/Institute of Prosthetic Dentistry and Dental Material Sciences, Medical University of Silesia in Katowice, 40-055 Katowice, Poland; aczelakowska@op.pl (A.C.); abialozyt@sum.edu.pl (A.B.); protstom@sum.edu.pl (J.K.); 2 Department of Materials Technology, Silesian University of Technology, 40-019 Katowice, Poland; Grzegorz.Chladek@polsl.pl; 3 Prosthodontic Department, Dental Institute, Jagiellonian University Medical College, 31-155 Krakow, Poland; andrzej.gala@uj.edu.pl (A.G.); magdalena.orczykowska@uj.edu.pl (M.O.); 4 Unit of Dental Propedeutics, Department of Conservative Dentistry with Endodontics, Division of Medical Sciences in Zabrze, Medical University of Silesia in Katowice, 40-055 Katowice, Poland; mskucha-nowak@sum.edu.pl

**Keywords:** older people, oral health, denture lining, long-term lining, soft lining materials, silicone-based lining, acrilic-based lining, gerodontology

## Abstract

The main functional feature of elastomeric soft linings materials is the ability to discharge loads in the tissues of the mucosa. As a result, there are fewer injuries to the mucosa and chewing ability increases. In addition, these prostheses are more comfortable in the patient’s opinion. To obtain the equal distribution of forces on the muco-bone basis and to reduce the traumatizing effect of the denture plate for patients using full dentures, soft lining materials can be used. Aim of the study: the aim of the work was a comparative laboratory study of ten materials used for soft lining of acrylic complete dentures. Methodology: Materials based on acrylates (Vertex Soft, Villacryl Soft, Flexacryl Soft) and on silicones (Sofreliner Tough Medium, Sofreliner Tough Medium, Ufi Gel SC, GC Reline Soft, Elite Soft Relining, Molloplast) were compared. Laboratory tests include tests of changes in Shore’a A hardness of soft lining material. The tests were conducted taking into account 90 day term aging in the distilled water environment based on the methodology presented in the European Standard ISO 10139-2. Results: For most silicone materials, only small changes in hardness were found in the range of 0.7 (Ufi Gel SC) to 3.3 (Sofreliner Tough Medium) on the Shore A scale. The exception was GC Reline Soft, for which a marked increase in hardness was noted. All materials based on acrylates were characterized by successive increase in hardness over time. However, in the case of the Vertex Soft material, the increase in hardness was relatively small (5.5 ShA).

## 1. Introduction

The use of acrylic full and partial removable dentures as well as postoperative dentures can be extremely difficult for patients. The most common problems reported by patients were those related to reduced functioning, such as difficulty in chewing, swallowing freely, and the occurrence of pain and discomfort [1,2,3,4,5,6]. Despite the correct construction of the prosthesis and its good fit, patients often report pain related to the transfer of occlusive forces to the prosthetic base. The long-term traumatizing effect of the prosthesis plate results mucosal injuries in the form of difficult to heal abrasions and bedsores [7,8,9,10]. In order to support prosthetic treatment and restore the physiological function of the stomatognathic system, soft relining materials can be used to cover the mucosal surface of the prosthesis [11]. The flexible lining layer affects the distribution of chewing forces on the mucoso-bone surface, better adjustment of the prosthesis, and also absorbs the pressure of the prosthesis, alleviating pain and facilitating the patient’s adaptation to the new dentures [12]. Complete denture retention is, in part, influenced by denture occlusion. Among denture users occlusal contact movements are performed randomly. These contacts may result from functional activity (swallowing) or parafunction (bruxism or clenching). A well balanced denture occlusion is intended to minimize the adverse effects of functional and parafunctional movements by widely distributing these forces to the denture bearing areas. The complete denture retention could also be improved by applying different methods, nevertheless, soft denture liners and denture adhesives are other modalities that could improve complete denture retention giving proper balance occlusion and in consequence equal distribution of the occlusal forces. Soft lining materials can help to evenly distribute the biting loads transferred onto soft tissues during chewing and to relieve the mucosa from high mechanical stress [13,14,15].

The use of soft materials for relining acrylic removable dentures may not only facilitate the use of dentures, but also completely eliminate pain sensations [12]. Moreover, clinical studies confirm that soft relining significantly improves the quality of life of patients [16]. According to Kucharski et al. the use of relining materials in the study group significantly shortened the adaptation time to new prostheses and significantly reduced the number of necessary corrections [17,18].

Similar results were obtained by Kimoto et al., who compared conventional acrylic dentures with dentures with a soft lining. The examinations were carried out in terms of the prosthetic basis conditions and the perception of pain [19]. The invaluable role of soft relining materials has also been demonstrated not only during the prosthetic rehabilitation of patients with difficult conditions of the prosthetic base, but also in the relief of alveolar processes after resection and implantation procedures, with clefts of the palate, during the treatment of inflammatory-fibrous hyperplasia, in patients suffering from scleroderma or in elderly people to accelerate the adaptation to new prostheses [17,18,19,20,21,22,23]. Currently, the most commonly used soft materials for long-term relining can be divided into two main groups due to their chemical composition: silicone and acrylic [16].

The main problem with the use of acrylic materials is their hardening directly caused by washing out of the plasticizers in the oral cavity environment. On the other hand, silicone materials do not contain plasticizers, and their elastic properties are due to their internal structure, thanks to which they are characterized by a generally stable hardness during use [24,25].

The materials used in dentistry are changing. Studies and research of their properties are current all the time. The knowledge of soft lining materials available on the market is valuable and helps to make a decision which one is the best to work with and that is why this aspect seems to be interesting to study further.

The assumption of the work was to compare according their hardness. The material, which increases in hardness during use, loses its therapeutic properties [24,26]. The appropriate degree of hardness and its stability over time is significant clinical importance and determines the period in which relining performs its therapeutic role. The undertaken research topic meets the unsolved problems of dental prosthetics and the social demand for properly functioning, and at the same time economical, solutions for dentures. The relining materials were assessed in terms of changes in hardness, which is important according the function of the dentures. The tested parameter directly determines the chewing efficiency and the quality of life of the patients. The hardness of acrylic and some silicone materials throughout the entire experiment period justify the need for benchmarking different products. The obtained results may contribute to the creation of an objective procedure for assessing the quality of the functioning of these materials based on laboratory and clinical criteria.

## 2. Materials and Methods

### 2.1. Laboratory Materials and Methods

Ten materials used for long-term soft relining of removable dentures were selected for the research. The materials include materials based on both: acrylates and silicones.

Tested silicone-based materials:

1. Sofreliner Tough M (Tokuyama, Taitou-ku Tokyo, Japan) is a soft material based on additional silicone. Binds AT room temperature. It is used in the indirect and direct method.
2. Sofreliner Tough S (Tokuyama, Taitou-ku Tokyo, Japan) is an A-silicone material. The product is qualified as a super soft material. The Sofreliner set includes pastes applied using an automix cartridge system and a primer used to bond with acrylic surfaces. The material binds at room temperature both in the direct and indirect method.
3. Mollosil Plus (Detax, Ettlingen, Germany) is a Type A silicone material that polymerizes at low temperature. It is available in automatic mixing containers when applied on a denture by direct and indirect methods.
4. Ufi Gel SC (VOCO, Cuxhaven, Germany) is a soft A-silicone material. The material is cured at room temperature, which makes Ufi Gel SC simple and quick to use. Relining can be done directly in the patient’s mouth and in the laboratory. The kit includes an automix cartridge system.
5. GC Reline Soft (GC, Tokyo, Japan) is a soft A-silicone-based relining material for long-term indirect and direct relining at room temperature. The material is available in automatic mixing cartridges.
6. Elite Soft Relining (Zhermack, Badia Polesine, Italy) is an additional silicone material for use in the dental office using the direct method and in the technical laboratory using the indirect method. Cross-linking of the material occurs at room temperature [26].
7. Molloplast B (Detax, Ettlingen, Germany) is a one-component silicone used in the indirect method. The material polymerizes at high temperature in a water bath or in the microwave.

Acrylic-based materials tested:

1. Vertex Soft (Vertex Dental, Soesterberg, The Netherlands) is a denture polymer that polymerizes under the influence of heat. Polymerization takes place by heating the material for 3 h at 70 °C, and then for 30 min at 100 °C.
2. Villacryl Soft (Zhermack, Badia Polesine, Italy) is an acrylic material that polymerizes at a high temperature—at 65 degrees under a pressure of 2.2 Bar for 30 min.
3. Flexacryl Soft (Lang Dental, Wheeling, IL, USA) is a flexible acrylic material used in the indirect method for relining denture plates.

Studies of Shore A hardness was carried out on the basis of the methodology presented in the European Standard ISO 10139-2 [27], but different aging times of samples in distilled water were used. The Shore A Hardness Scale measures the hardness of flexible mold rubbers that range in hardness from very soft and flexible, to medium and somewhat flexible, to hard with almost no flexibility at all. Semi-rigid plastics can also be measured on the high end of the Shore A Scale. The hardness of materials is determined by the Shore method using an instrument called a durometer. Durometer measures the penetration depth in the material created by a given force on a standardized presser foot. This depth is dependent among others on the hardness of the material. The measurement requires applying the force in a consistent manner and measuring the hardness. The spring-pushed indenter presses into the material, whereby a balance is established between the spring pressure and the material response. Once equilibrated, the pointer stops in the appropriate range from 0 to 100 Shore units (maximum hardness—zero penetration) [27].

Hardness measurements were made one hour after their execution and after 24 h, and then after 7, 28, 60 and 90 days of soaking the samples in distilled water. The aging times of the samples in distilled water were selected in accordance with the methodology presented in the European Standard ISO 10139-2. All samples of materials for testing were made in a specially designed steel form covered with a lid with a diameter of 40 mm and a depth of 6 mm, so that each of the samples had the same size and shape. The polymerization of samples from all materials was carried out according to the manufacturers’ recommendations. The analyzed materials were mixed in the proportions recommended by the manufacturer using a dispenser with a mixing tip or manual, then the material was applied to the form. In order to prevent damage to the samples during removal from the form, additional spacers made of 100 µm thick polyester foil were used. Three samples (for correctness and repeatability) were made of each material and placed in distilled water at a temperature of 37 ± 1 °C for 24 h, 7, 28, 60 and 90 days. Shore A hardness was measured within 2 ± 1 min of removing each sample from the bath, and the measurement was read 5 s after loading. The hardness for each of the samples was measured at five different points not less than 5 mm from the edge of the sample and 2 mm from each other. After measurement, the sample was immediately placed back in the water until the next measurement time.

An ethical approval of the Bioethical Commission of the Medical University of Silesia in Katowice was not required.

### 2.2. Statistical Analysis

Statistical analysis using the PQStat ver. 1.6.6.204 (PQStat Software, Poznan, Poland) was made. The data obtained during laboratory tests were used for calculations aimed at finding statistically significant relationships and comparing the results of individual materials used for long-term, soft acrylic dentures relining. The level of significance was α = 0.05.

The test results were analyzed of variance (ANOVA) for one-factor systems (α = 0.05) with a possible F * correction (Brown–Forsythe), when the assumption of equal variance was not met. The tests were preceded by checking the assumption of homogeneity of variance with Levene’s test. In case of rejection of the null hypothesis, determined by the analysis of variance of the lack of equality between the means, the differences between the means of the individual groups were examined using the post-hoc honestly significant difference (HSD) Tukey test. In this way, the difference and equality between the n-means were checked

The obtained results were summarized and presented in the form of tables and figures. In the figures, the same letters denote the mean values, which did not differ in a statistically significant manner.

## 3. Results

Requirements according to ISO 10139-2 [27] in terms of hardness by the different materials in the context of their long-term use is presented in Table 1. The requirements of the standard in this respect were not met by the Flexacryl Soft material, for which the hardness values exceeded the acceptable criteria. In addition, Sofreliner Tough Soft and Villacryl Soft could be classified as super soft materials, and the rest of the materials as soft.

**Figure 1:**
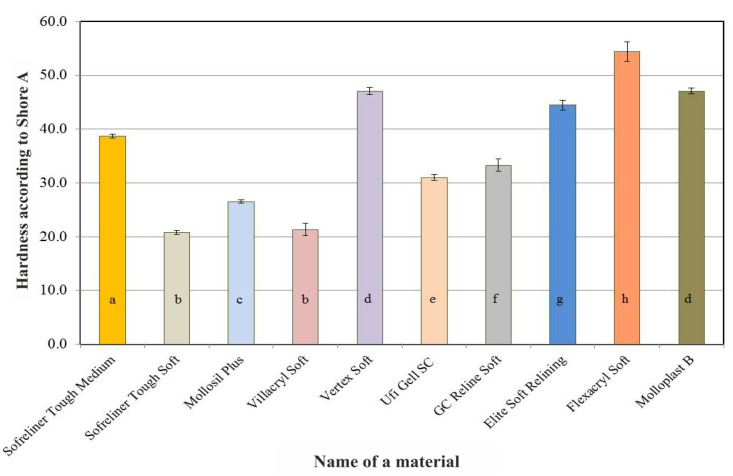
Average Shore A hardness values of the tested relining materials after 24 h of conditioning the samples in distilled water. The same letters denote mean values which do not differ statistically significantly.

A summary of the results of the Shore A hardness tests of the lining materials carried out after 24 h of conditioning in distilled water is shown in Figure 1.

A statistically significant effect of the lining material used (*p* < 0.0001) on the hardness values was found (Table 2). Acrylic Villacryl Soft and silicone Sofreliner Tough Soft were characterized by the lowest hardness values, which did not differ in a statistically significant manner. The silicone materials were characterized by a significant diversification of the average hardness values, ranging from 20.8 to 47.1 units on the Shore A scale. At the same time, all of the seven tested materials differed from each other in this respect.

**Figure 2:**
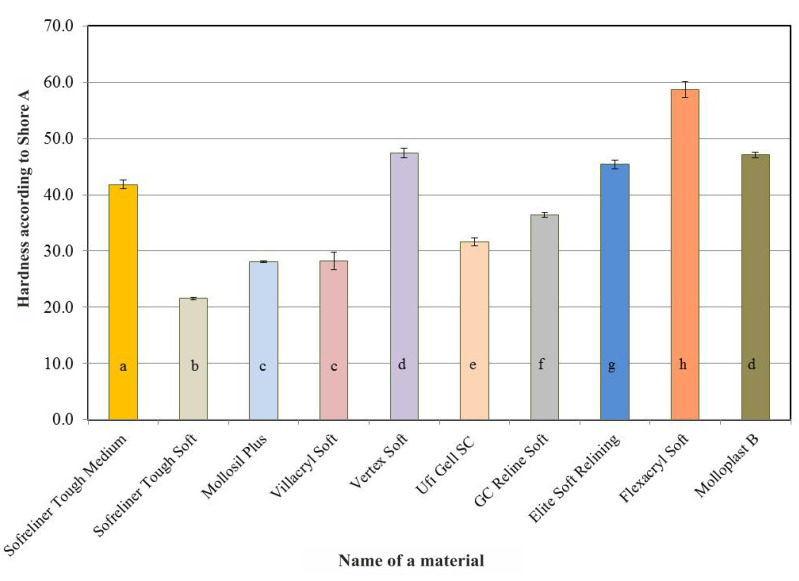
Average Shore A hardness values of the tested relining materials after seven days of conditioning the samples in distilled water. The same letters denote mean values which do not differ statistically significantly.

**Figure 3:**
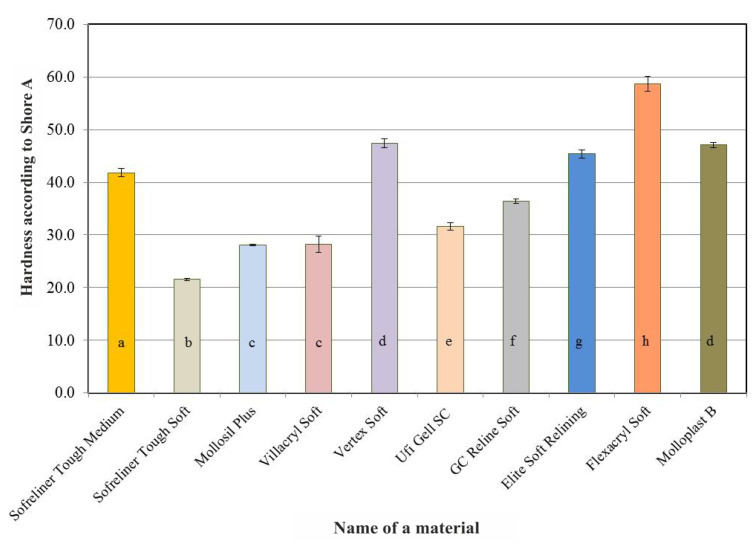
Average Shore A hardness values of the tested relining materials after 28 days of conditioning the samples in distilled water. The same letters denote mean values which do not differ statistically significantly.

**Figure 4:**
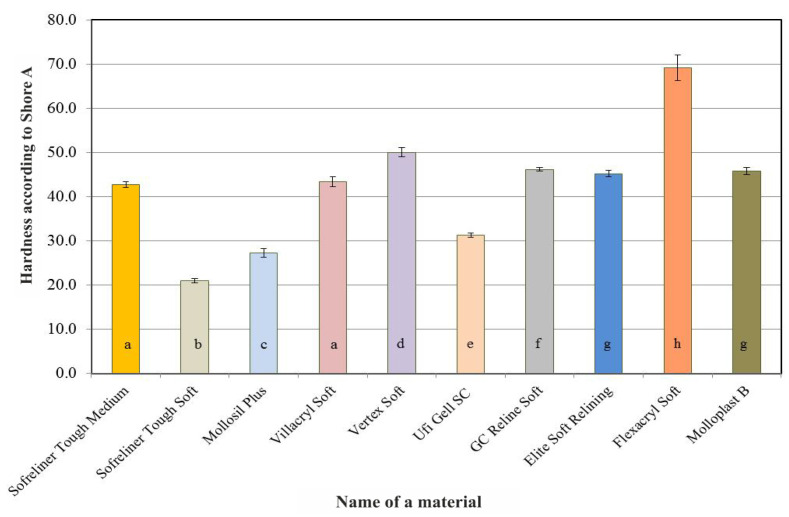
Average Shore A hardness values of the tested relining materials after 60 days of conditioning the samples in distilled water. The same letters denote mean values which do not differ statistically significantly.

**Figure 5:**
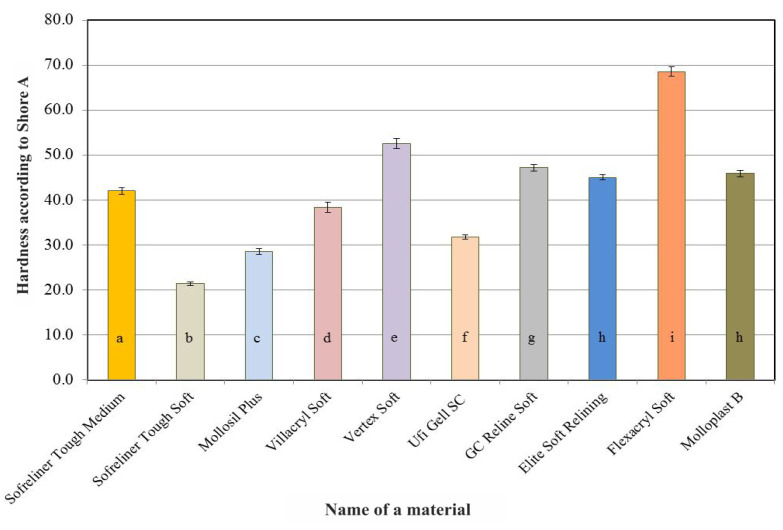
Average Shore A hardness values of the tested relining materials after 90 days of conditioning the samples in distilled water. The same letters denote mean values which do not differ statistically significantly.

Acrylate-based materials: Vertex Soft and Flexacryl Soft showed higher initial hardness than silicone materials. A summary of the results of Shore A hardness tests for the lining materials carried out after seven days of conditioning in distilled water is shown in Figure 2. As in the case of the initial values, Vertex Soft and Flexacryl Soft acrylic materials (average hardness values: 47.4 and 58.7) showed greater hardness than silicone materials, whose average hardness value did not exceed 47.1 on the Shore A scale. The differences in average hardness values after 28 days (Figure 3), 60 days (Figure 4) and 90 days (Figure 5) of conditioning were similar. A visible increase in the hardness of acrylic materials and an increase in the hardness of some silicone materials throughout the entire experiment period indicated the necessity to carry out statistical analyzes in terms of the influence of the conditioning time on hardness.

The performed statistical analyzes showed statistically significant changes in the degree of hardness of all the lining materials during the three months of conditioning samples in distilled water, in relation to the initial values (Table 3). For most silicone materials reported only slight changes in hardness within the range of 0.5 (Elite Soft Relining) to 3.4 (Sofreliner Tough Medium) units on the Shore A scale. This demonstrates the high hardness stability of these materials. The exception was the GC Reline Soft material, for which a significant increase in hardness was noted during the first 28 days of conditioning.

## 4. Discussion

The multitude and variety of materials for soft relining currently available on the market may pose a problem of their rational selection for specific clinical situations. The key issue is an objective analysis of selected mechanical and functional properties of flexible materials commonly available. Knowing the characteristics of the product, it is possible to select precisely the material depending on the specific conditions in the oral cavity, the individual needs of the patient and therapeutic purpose. The author’s own research showed changes in the degree of hardness measured by the Shore A method, and the obtained results were checked in terms of meeting the requirements of ISO 10139-2. Flexacryl Soft did not meet the hardness requirements for soft lining materials. Its average hardness values were 54.4 ShA after 24 h and 58.7 ShA after 28 days, exceeding the acceptable criteria. In the light of the obtained results, it is necessary to consider the sense of using this material in the context of soft denture relining.

The results of own research show that the average hardness values of silicone materials after 24 h and after three-month conditioning in distilled water were similar. In most silicone materials, the changes in hardness ranged from 1.1% (Elite Soft Relining) to 8.1%, which is not significant from the clinical point of view. Therefore, it can be concluded that silicone materials are characterized by high stability of hardness over time, which is confirmed by the results of studies available in the literature [28,29,30,31,32,33,34,35]. The exception among the tested silicone materials was GC Reline Soft, which showed a large increase in hardness, a particularly clear increase was recorded during the first 28 days. During three months of observation, its average hardness value increased by 29% reaching the value of 47.2 ShA. Some publications show that there are silicone-based materials on the dental market that show much larger changes in hardness over time. Kim et al. [28] and Iwaki et al. [29] showed changes in the hardness of the super-soft GC Reline Ultrasoft material during 28 days of observation, which amounted to 63%.

The test results showed a gradual increase in hardness over time for all acrylate-based materials. Such significant differences in the changes in the hardness of silicone and acrylic materials may result from their chemical structure and basically “rinsing out” the plasticizers in the oral cavity environment. The desired hardness of silicone materials is obtained by their degree of cross-linking or by adding suitable fillers. On the other hand, initially low hardness values of acrylic materials are obtained mostly by the use of plasticizers, which over time are washed out or dissolved in an aqueous medium and at a constant temperature of 37 °C. Plasticizer molecules can separate polymer chains, which lowers the glass transition temperature and makes the material softer. However, plasticizers are not permanently connected to the resin, that is why they leach out of it, and causes the huge changes in their mechanical properties. During the three-month observation, the hardness of the tested acrylic materials increased from 20.7% (Flexacryl Soft) to 44.4% (Villacryl Soft). In the case of Vertex Soft material, the increase in hardness was relatively small and amounted to 10.5%. These results clearly show that acrylic materials are much less stable in terms of hardness over time than silicone materials. The obtained results are confirmed in numerous scientific reports and publications on the hardness of the lining materials [28,29,30,31,32,33,34,35].

Mese et al. [30] tested two silicones, Mollosil Plus and Molloplast B, and two acrylics, Vertex Soft and Coe Comfort. After six months of observation, the hardness of silicones increased to 18%, and of acrylics even to 100%. Similar studies were independently conducted by Canay et al. [31] and after six months of conditioning the samples in distilled water, similar results were obtained. Kim et al. [28] investigated the Shore A hardness of one acrylic-based relining material and six silicone materials. After 28 days of aging of the samples in an aqueous environment, the hardness of the silicones increased from 5% (Mucosoft) to 63% (GC Reline Ultrasoft), while the hardness of the Durabase acrylic increased by 34%. Polyzois et al. [32] investigated two acrylic materials: the room temperature polymerizing EverSoft and the hot polymerizing Super-Soft. The samples were conditioned in distilled water for a year. During the first month, the hardness of the Super-Soft material changed by 8% and its value stabilized. In the case of EverSoft, these changes were 120% after the first month and 150% the following, and then also plateaued. However, it should be noted that the initial hardness of Super-Soft was much higher than that of EverSoft.

## 5. Conclusions

Based on the research, it can be concluded that most of the tested silicone materials show a stable degree of hardness after 24 h and 7, 28, 60 and 90 days. However, in the case of one silicone material—GC Reline Soft, a large increase in hardness was observed, a particularly marked increase was noted after 28 days. All the tested acrylic-based materials showed a gradual increase in hardness during the test.

## Figures and Tables

**Table 1:** Comparison the degree of meeting the requirements of ISO 10139-2 for the test materials in the light of the criteria for classification of soft relining material as soft or super soft.

Trade Name of the Material	Conditioning Time in Distilled Water			
	24 h		28 Days	
	Shore’a A ≤ 25	25 < Shore’a A ≤ 50	Shore’a A ≤ 35	Shore’a A ≤ 55
Sofreliner Tough Medium	N	Y	N	Y
Sofreliner Tough Soft	Y	Y	Y	Y
Mollosil Plus	N	Y	N	Y
Villacryl Soft	Y	Y	Y	Y
Vertex Soft	N	Y	N	Y
Ufi Gel SC	N	Y	N	Y
GC RELINE Soft	N	Y	N	Y
Elite Soft Relining	N	Y	N	Y
Flexacryl Soft	N	N	N	N
Molloplast B	N	Y	N	Y

N—no, Y—yes.

**Table 2:** Influence of the type of relining material used on the Shore A hardness.

Conditioning Time	Sum of Squares	Degrees of Freedom	Root Mean Square	Value of the Fisher Statistic	*p*
24 h	18,439,724	9	2,048,858	2,302,482	<0.0001
7 days	17,608,458	9	1,956,495	2,605,055	<0.0001
28 days	24,360,182	9	2,706,687	1,256,763	<0.0001
60 days	24,319,921	9	2,702,213	1,790,562	<0.0001
90 days	23,911,916	9	2,656,880	3,930,129	<0.0001

**Table 3:** Influence of the conditioning time of material samples on the hardness of the underlying materials.

Material	Sum of Squares	Degrees of Freedom	Root Mean Square	Value of the Fisher Statistic	*p*
Sofreliner Tough Medium	157,845	4	39,461	74,141	<0.0001
Sofreliner Tough Soft	6693	4	1673	10,642	<0.0001
Mollosil Plus	37,971	4	9493	24,435	<0.0001
Villacryl Soft	4,469,218	4	1,117,304	767,379	<0.0001
Vertex Soft	306,887	4	76,722	75,161	<0.0001
Ufi Gel SC	5024	4	1256	3386	0.0137
GC Reline Soft	2,547,227	4	636,807	991,966	<0.0001
Elite Soft Relining	7194	4	1799	2583	0.0445
Flexacryl Soft	2,953,463	4	738,366	120,034	<0.0001
Molloplast B	2,547,227	4	636,807	13590	<0.0001

## Data Availability

The data presented in this study are available from the corresponding authors.
